# Determinants of Direct Support Professionals’ Mealtime Experiences in an Israeli Long-Term Care Facility for Residents with Intellectual and Developmental Disabilities

**DOI:** 10.3390/nu18091388

**Published:** 2026-04-28

**Authors:** Rinat Avraham, Leah Levy Ya’akobov, Natalia Kondelis, Odeya Cohen

**Affiliations:** 1 Department of Nursing, Recanati School of Community Health Professions, Faculty of Health Sciences, Ben-Gurion University of the Negev, Beer-Sheva 8410501, Israel; leahlevy84@gmail.com (L.L.Y.); natalik.moria@gmail.com (N.K.); odeyac@bgu.ac.il (O.C.); 2 Aleh Moria, Rehabilitation Center for People with Disabilities, Levinson St. 3, Gedera 7057003, Israel

**Keywords:** Direct Support Professionals (DSPs), feeding, Intellectual and Developmental Disabilities (IDD), long-term care, mealtime experience

## Abstract

**Background**: Malnutrition is a universal challenge in long-term care, significantly affecting vulnerable populations. Residents with Intellectual Developmental Disability (IDD) rely heavily on Direct Support Professionals (DSPs) for assisted feeding. Understanding DSP’s mealtime experiences is essential for improving nutritional care and well-being. **Objective**: To examine multilevel factors associated with DSPs’ mealtime experiences. **Methods**: This exploratory cross-sectional case study used a survey administrated to DSPs working in a long-term residential setting. Statistical analyses examined the associations between multilevel factors and DSP’s positive and negative mealtime experiences. **Results**: The sample included 46 DSP’s (98% women) from a single facility in Israel. Although DSPs reported high levels of positive feelings and satisfaction with their daily work efficacy, negative feelings were significantly associated with some organizational, environmental and resident-related factors. Negative feelings were higher among DSPs caring for residents who use wheelchairs compared to those working with residents who do not use wheelchairs (t = −2.99, *p* < 0.01). Negative feelings were negatively associated with institutional support (r = −0.49, *p* < 0.001), and perceived accessibility and adaptability of the environment (r = −0.46, *p* = 0.001), and showed a more modest association with communication with residents (r = −0.38, *p* = 0.01). DSPs’ seniority, education level, and prior feeding-related training were not significantly associated with mealtime experience. **Conclusions**: The findings highlight that negative mealtime experiences among DSPs are associated with organizational, environmental, and resident-related factors, rather than with individual DSP’s characteristics. Policy and practical adjustments to address mealtime experiences for residents with IDD are suggested.

## 1. Introduction

Appropriate nutrition is a fundamental determinant of health, functional ability, and quality of life [1,2], particularly among populations who rely on others for assistance with daily activities [3]. Individuals with Intellectual and Developmental Disabilities (IDD) are at increased risk of nutritional inadequacy due to physical, cognitive, and sensory impairments that affect eating performance [4,5]. In long term residential living facilities, where individuals with IDD receive daily support, mealtime assistance is primarily delivered by Direct Support Professionals (DSPs) and constitutes a central component of routine care. Despite the clinical and functional importance of feeding practices, limited attention has been given to the experiences of DSPs who provide daily mealtime support. Understanding the multilevel factors that are associated with DSPs’ mealtime experiences is therefore essential for advancing person-centered care and improving nutritional support in long-term residential living facilities.

## 2. Background

IDDs are characterized by significant limitations in intellectual functioning and adaptive behavior originating during the developmental period, and may co-occur with conditions such as intellectual disability, autism spectrum disorder, and cerebral palsy [6]. The global prevalence of IDD is estimated at approximately 1.74% of the total population, affecting over 107 million individuals as of 2019, with rates slightly higher in males (1.42%) than females (1.37%) [7]. Individuals with IDD experience higher rates of chronic physical conditions, mental health disorders, and eating and swallowing difficulties compared with the general population [5,8].

Eating is a core activity of daily living (ADL) and a key determinant of health and functioning status [1,2,9]. In residential settings, mealtimes represent structured daily interactions that combine physical assistance with social exchange and emotional engagement. Among individuals with IDD, eating performance is often compromised due to physical, sensory, and cognitive limitations, resulting in reduced food and fluid intake and increased reliance on caregiver support [4,10]. Beyond meeting nutritional requirements, mealtimes function as socially embedded institutional practices that shape daily routines and opportunities for participation, relational engagement, and social inclusion [11].

Feeding individuals with IDD is a complex routine with significant health implications [5]. Safe feeding requires attention to positioning, pacing, food temperature, texture modification, and monitoring for signs of distress, particularly among individuals with dysphagia or severe mobility limitations [5,10]. Feeding practices are also shaped by system-level conditions such as staffing levels, workload, and time constraints [12]. Caregivers frequently balance dietary and medical recommendations with the need to assist multiple residents simultaneously, which may limit person-centered support [13,14]. Environmental characteristics including noise, lighting, seating arrangements, and distractions, also influence feeding processes and eating performance [15]. Together, these evidence underscore feeding as an outcome of interacting health, organizational, and environmental systems factors [15].

DSPs are the primary workforce in residential facilities that are responsible for residents’ nursing care, assisting with essential activities of daily living, including feeding [16,17]. In Israel, these facilities operate within the national welfare system and provide 24 h support for individuals unable to live independently in the community [18]. DSP training typically occurs through institution-specific and on-the-job programs rather than standardized education, resulting in variability in competencies and experience [14].

DSPs’ care during mealtimes is influenced by interacting individual, interpersonal, organizational, and environmental determinants [19]. At the individual level, the severity of intellectual disability, physical mobility, and sensory or communication impairments determine the complexity of feeding support required [10,20]. Effective interpersonal caregiver–resident communication has been associated with reduced stress and improved satisfaction during meals [14]. Organizational factors such as staffing shortages, high workload, time pressure, and limited training may restrict caregivers’ ability to provide individualized support and contribute to stress and frustration [13,14]. Røstad-Tollefsen et al. [12] emphasize that nutritional care is embedded within systemic structures that shape its delivery, including competing priorities, insufficient knowledge, and ethical tensions within regulatory frameworks. Environmental characteristics of the dining setting may further facilitate or hinder effective feeding interactions [11].

Existing research on feeding in residential settings for individuals with IDD has largely focused on clinical and nutritional outcomes, including dysphagia management, feeding safety, and dietary adequacy [5,10]. Although studies acknowledge the central role of DSPs in shaping nutritional practices (e.g., [12,19], limited evidence exists regarding DSPs’ experiences during routine feeding interactions and how multilevel determinants shape these experiences.

**Figure 1:**
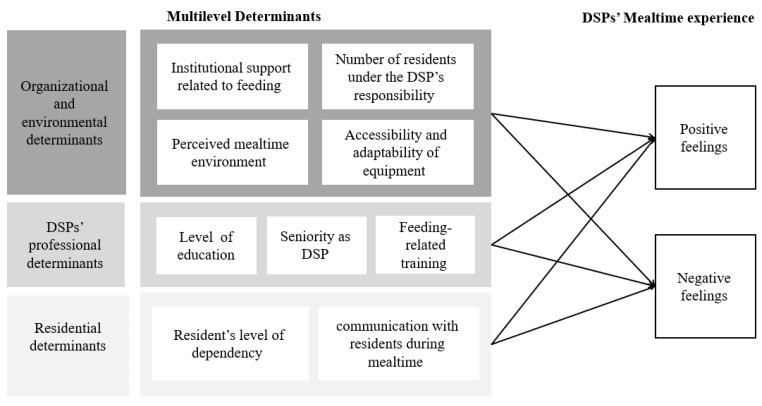
Conceptual Framework of the Study.

Therefore, the present exploratory study aimed to examine DSPs’ mealtime experiences in a long-term residential living facility for individuals with IDD, and to investigate their associations with organizational, environmental, individual, and residential determinants. Figure 1 presents the conceptual framework of the study.

### Study Hypotheses

**H1.**  *Organizational and environmental determinants.*

**H1a.**  *DSPs that feed more than five residents during mealtimes will present higher negative feelings and lower positive feelings compared to those feeding less residents.*

**H1b.**  *Stronger perception of institutional support will be associated with higher positive feelings and lower negative feelings.*

**H1c.**  *Higher mealtime environmental scores will be associated with higher positive feelings and lower negative feelings.*

**H1d.**  *Higher administrative accessibility and adaptability will be associated with higher positive feelings and lower negative feelings regarding feeding.*

**H2.**  *DSPs’ professional determinants.*

**H2a.**  *Higher seniority, level of education and formal training will be associated with higher positive feelings and lower negative feelings regarding feeding.*

**H3.**  *Residential determinants.*

**H3a.**  *DSPs working with individuals who use wheelchairs will present higher negative feelings and lower positive feelings compared to DSPs working with individuals who don’t use wheelchairs.*

**H3b.**  *Higher scores in communication with residents during mealtimes will be associated with lower negative feelings and higher positive feelings.*

## 3. Materials and Methods

This exploratory study employed a cross-sectional observational design among DSPs in a long-term residential living facility for people with IDD. The study followed the STROBE guidelines.

### 3.1. Setting and Sampling

This study employed a continuous sampling method in a single long-term residential living facility for people with IDD in Israel. This facility houses approximately 100 residents aged 16–45, living in small group homes organized by gender and level of functioning, and is staffed by approximately 65 DSPs, about 97% of them are women. All residents have a primary diagnosis of IDD, often accompanied by comorbid conditions such as autism spectrum disorder, epilepsy, and behavioral challenges. Participation in the study was voluntary. Inclusion criteria included DSPs who work at the institution for at least six months and participate in feeding tasks during their worktime. Participants who do not speak Hebrew were excluded.

### 3.2. Data Collection

Data were collected in March 2023 using self-administered questionnaire. The questionnaire was completed in a pen-and-paper format. Participants who were able to read and write independently completed the survey on their own. For participants who needed help, a trained research assistant read the items aloud and recorded the participants’ responses. To mitigate interviewer influence, research assistants received prior training on standardized administration procedures, including maintaining neutrality and minimizing interpersonal influence during data collection. Participants were also informed that their responses were confidential and that there were no right or wrong answers. Questionnaires were completed anonymously in the residential facility during working hours at times that minimized disruption to routine care (e.g., during quieter periods or at the end of shifts). Participation was voluntary, and participants were informed that they could decline or withdraw at any time without consequences. Research assistants emphasized the independent nature of the study, and no managerial pressure was applied.

### 3.3. Measurement

Data were collected using a self-report questionnaire developed in 2020 by Keren Shalem to examine the feeding experiences of DSPs working with individuals with IDD. The instrument was previously used in the evaluation of the program “A Meal is More Than Food”, an initiative aimed at enhancing the eating experience through assistive technologies and adaptive devices among individuals with IDD and their DSPs [21].

The questionnaire consisted of two sections:

1. Socio-demographic and professional characteristics, including age, gender, religion, level of education, years of professional experience, assisting residents who use wheelchairs, number of residents under the participant’s responsibility, and prior training in feeding skills.
2. Attitudes towards eating and feeding assessed by 22 items capturing DSPs’ perceptions of the mealtime experience within the facility. The grouping of items into domains was conceptually driven, based on the theoretical framework underlying the intervention in the program mentioned above. The items were organized into six domains: (1) mealtime environment, (2) communication with residents, (3) accessibility and adaptability of the environment, (4) institutional support, (5) negative feelings related to job efficacy, and (6) positive feelings related to job efficacy. Items were rated on a five-point Likert scale (1 = strongly disagree; 5 = strongly agree).

### 3.4. Study Variables

The primary outcome variable, mealtime experience, was operationalized through two dimensions: negative feelings such as stress and workload (five items, e.g., During feeding, I think about other tasks I need to complete before the end of my shift), and positive feelings such as satisfaction derived from feeding activities (three items, e.g., I feel satisfied when the resident enjoys the food). Independent variables were organized according to the multilevel framework guiding the study: At the organizational and environmental level, we measured number of residents under the DSP’s responsibility (≤5 vs. >5), perceived mealtime environment (four items, e.g., The dining room is pleasant and inviting, and I feel comfortable sitting in it), accessibility and adaptability of the eating equipment (four items, e.g., The dining tables are adapted to the needs of the residents), and institutional support (two items, e.g., I am involved in decision-making related to residents’ feeding). At the DSP’s individual level, we assessed DSP education, seniority, and prior feeding-related training. At the resident level we measured communication with residents during mealtime (three items, e.g., There is conversation between me and the resident during feeding) and whether the DSP assist residents who use wheelchairs as a proxy to resident’s level of dependency (dichotomous variable).

### 3.5. Data Analysis

Analyses were performed using IBM SPSS Version 29. For between-group comparisons we used independent *t*-tests. Pearson’s correlation coefficients were performed to examine the relationships between main study variables. Prior to analysis, assumptions for parametric tests were assessed. Normality of continuous variables was evaluated using skewness and kurtosis values, and homogeneity of variance was examined for *t*-tests. Linearity was assessed for correlation analyses. No substantial violations were identified. Significance level was set at α < 0.05.

### 3.6. Ethical Considerations

The study was approved by the Institutional Review Board of the Faculty of Health Sciences at Ben-Gurion University of the Negev (Approval No. 02-2023) on 2 December 2023. Additional approval was obtained from the Medical and Administrative Office of the participating residential facility. All participants received a detailed explanation of the study aims, procedures, and data confidentiality prior to participation. Those who agreed to participate provided written informed consent.

## 4. Results

About 70% of the DSPs in the residential facility participated in the study (*n* = 46), of which 97.8% were women and 63% identified as Jewish. More than half (54.3%) were between 42 and 65 years old, while the remaining participants were younger (18–41 years). In terms of religious affiliation, 45.6% described themselves as religious. Most DSPs (80.4%) had completed 12 years of education, and an additional 15.2% held a bachelor’s degree.

Regarding seniority in working with individuals with IDD, approximately half of the DSPs had up to five years of experience, 21.7% reported 6–10 years, and the remainder had 11–25 years. Most DSPs (76.1%) typically worked with residents who walk independently, whereas the rest worked with residents who use a wheelchair. A majority (60.9%) reported feeding 3–5 residents per meal, while others fed 6–8 residents. About half of the DSPs had participated in a two-hour educational session on feeding practices during their employment in the residential living facility.

Table 1 presents the descriptive statistics and internal consistency coefficients of the attitude’s domains. Positive feelings were rated very highly (M = 4.64, SD = 0.48) with a restricted range, indicating minimal variability. Negative feelings demonstrated medium-to-high scores (M = 3.91, SD = 0.69). Internal consistency ranged from acceptable to good (α = 0.63–0.89).

### Testing the Hypotheses

In testing the associations between organizational and environmental determinants and mealtime experiences (H1a–H1d), we found no significant differences between DSPs that feed more than five residents during each meal to those who feed five residents or less. Perceptions of accessibility and adaptability were negatively associated with negative feelings (r = −0.46, *p* = 0.001). Finally, institutional support was negatively associated with negative feelings (r = −0.49, *p* < 0.001).

Regarding H2, no significant association was found between participants’ seniority and positive or negative feelings about job efficacy. In addition, no significant differences were observed between participants with a high school education and those with a professional or academic degree. Finally, no significant differences were found in terms of positive feelings and negative feelings between participants received formal training in feeding individuals with IDD to those did not receive formal training.

To test the hypotheses at the residential level (H3a and H3b), DSPs working with wheelchair users reported significantly more negative feelings related to job efficacy compared to those working with residents who do not use wheelchairs (M = 4.42 vs. 3.75, t = −2.99, *p* < 0.01, Cohen’s d = 0.64). No significant differences were found between the groups in positive feelings about job efficacy. DSPs working with wheelchair users also reported lower levels of perceived institutional support (M = 2.55 vs. 3.84, t = 4.87, *p* < 0.001, Cohen’s d = 0.77). In addition, communication with residents during mealtime was negatively associated with negative feelings (r = −0.38, *p* = 0.01), indicating that better communication was linked to fewer negative emotional experiences. Communication with residents score was strongly positively associated with perceptions of institutional support (r = 0.59, *p* < 0.001) and accessibility and adaptability (r = 0.74, *p* < 0.001).

## 5. Discussion

This study explored the factors associated with DSPs’ mealtime experiences in a long-term residential facility for individuals with IDD. Overall, DSPs reported high levels of positive feelings and satisfaction with work efficacy despite moderate levels of negative feelings related to stress and workload. Notably, only negative feelings were significantly associated with contextual and resident-related factors. In contrast, positive feelings were not significantly associated with individual, resident-related, organizational, or environmental variables.

### 5.1. Positive and Negative Feelings

The coexistence of positive and negative emotional experiences among caregivers is well documented. Stamm [22] described the Compassion Satisfaction and Compassion Fatigue framework, which conceptualizes caregiving work as encompassing both rewarding and taxing emotional dimensions that may coexist rather than represent opposite ends of a single continuum. Similarly, research indicates that paid caregivers may experience both positive and negative effects of caregiving simultaneously; caregivers of individuals with IDD, for example, have been shown to report elevated levels of stress and burnout alongside sustained commitment and compassion [23]. In the context of nutritional and feeding support, mealtime assistance for individuals with cognitive impairments has been described as a complex caregiving activity that is physically demanding, emotionally intensive, and highly time-sensitive [24,25]. Røstad-Tollefsen et al. [12] found that staff working with adults with IDD experience considerable strain associated with workload, organizational constraints, and the complexity of residents’ needs. At the same time, Avraham et al. [26] demonstrate that institutional mealtimes are relationally and symbolically meaningful, indicating that feeding may generate positive emotions grounded in interpersonal connection and professional commitment, despite its practical demands. Similarly, Özdemir et al. [27] emphasize the relational complexity of feeding in IDD contexts, showing that carers continuously negotiate between promoting healthy eating and respecting residents’ autonomy. Negative emotions may reflect structural and environmental constraints, positive feelings may stem from the relational significance and moral commitment embedded in mealtime care. The lack of significant associations involving positive feelings may be attributed to the high mean scores and limited variability observed in this construct, suggesting a potential ceiling effect or social desirability bias. This is particularly relevant considering the presence of research assistants during data collection, which may have constrained the ability of the measure to detect meaningful differences or associations.

### 5.2. Interacting Individual, Interpersonal, and Organizational Influences

Beyond the emotional dimensions of caregiving, the present findings provide additional empirical support for the ecological framework which conceptualizes feeding experiences as shaped by interacting individual, interpersonal, organizational and environmental determinants [12,19]. In the current study, individual characteristics such as seniority, education, and formal training were not significantly associated with caregivers’ emotional experiences, whereas interpersonal and organizational factors demonstrated consistent relationships with negative feelings and perceived institutional support. Communication with residents, perceived environmental accessibility and institutional support were associated with lower negative feelings. These findings suggest that emotional experiences during feeding are reflected more by context and relationships than by individual professionalism or education. This is aligned with prior research indicating that nutritional care in residential settings is shaped by structural and environmental conditions, including workload, staffing constraints, and institutional policies [13,14]. A recent systematic review underscores how organizational support is linked to caregiver well-being in healthcare settings, with supportive structures reducing stress and protecting mental health, whereas lack of support exacerbates negative emotional experience [28]. Feeding in residential IDD settings appears to be embedded within a system of interactions and institutional resources, where environmental support and communicative quality may buffer emotional strain more effectively than individual characteristics alone [3].

In this study, institutional support specifically reflected staff involvement in feeding-related decision-making and the availability of professional support when challenges arise. These findings suggest that fostering participatory work environments and ensuring accessible support mechanisms for DSPs may be particularly important for reducing negative emotional experiences during mealtimes. The lack of significant association between formal training and emotional experience may reflect the fact that a brief, non-standardized training session (e.g., a single two-hour workshop) does not sufficiently address the structural and relational demands inherent in feeding individuals with complex needs.

### 5.3. Institutional Support and Resident Complexity

We found that DSPs working with highly physically dependent residents reported negative feelings and job experience with lower perceived institutional support. Feeding individuals with complex physical needs often requires sustained physical assistance and close monitoring, which increases task demands [5] and may be shaped by organizational conditions such as staffing and workload [12]. On the other hand, this increased burden may also reflect ergonomic and environmental challenges associated with feeding individuals with complex conditions, such as non-adjustable furniture, positioning constraints, or limited access to assistive equipment. Research has shown that feeding in developmental disability contexts is frequently experienced as stressful and emotionally demanding rather than purely routine tasks [29]. Although this study focused on family caregivers, it highlights the inherently challenging nature of feeding when communication difficulties and behavioral issues are present. Support work in intellectual disability services has been described as relational and requiring professional judgment in everyday interactions [30]. In high-dependency feeding situations, attention may necessarily focus on safety and task completion, potentially leaving less space for mutual engagement during meals. Environmental factors may further contribute to caregiver strain alongside resident dependency. Organizational policy in long-term care settings may benefit from addressing communication and relational strategies, particularly among complex individuals, considering both the additional demands for DSPs and the need for environmental design adjusted for residents who require extensive physical assistance. Institutional support may also play a mediating role in the relationship between resident complexity and DSP’s stress. This pathway warrants further investigation.

### 5.4. Implications for Practice

The present findings underscore the importance of addressing mealtime support through a system-oriented lens. First, there is a need for clear organizational policies, accessible supervision, and structured guidance around feeding practices. Second, environment of care emerged as a key factor, suggesting that investment in adaptive equipment, ergonomic arrangements, and accessible design may directly reduce caregiver strain. Third, communication with residents served as a relational buffer. Institutional cultures that promote communicative engagement during meals may simultaneously support resident participation and caregiver well-being. Fourth, the negative association between working with wheelchair users and feeding efficacy supports the need for structured support when feeding residents with mobility impairments. Finally, improvements should not rely solely on brief, one-time educational interventions.

### 5.5. Study Limitations

This study has several limitations that should be acknowledged. First, the relatively small sample size (*n* = 46) may limit the generalizability of the findings to broader or more diverse populations. It also limits the statistical power of the analyses and restricted the ability to conduct more advanced inferential modeling, while the reliance on univariate analyses limits the ability to account for potential confounding between variables and to fully capture the complexity of multilevel relationships. In addition, the study sample was predominantly female and drawn from a single facility using a continuous sampling approach, which may limit the generalizability of the findings. However, the gender distribution reflects the composition of the direct support workforce in this setting. Moreover, residential facilities for individuals with IDD in Israel operate under national supervision by the Ministry of Welfare and Social Affairs and follow standardized regulatory and training frameworks, which may partially support the contextual relevance of the findings to other long-term residential facilities.

Second, data were collected through interviewer-administered questionnaires rather than fully self-administered surveys. Some participants were not native Hebrew speakers and had limited reading proficiency, which could have affected their ability to independently understand written survey items. While interviewer administration helped ensure comprehension and accurate completion of the questionnaire, this approach may have increased the risk of social desirability bias, as participants may have provided responses perceived as professionally appropriate. This may be particularly relevant for the high levels of reported positive feelings, which showed limited variability and may partly reflect socially desirable responding in a workplace context, where participants may have felt expected to express positive attitudes toward their work. However, this approach was necessary to ensure inclusion of participants with varying levels of literacy.

Finally, resident dependency was operationalized using wheelchair use as a proxy measure. While this indicator reflects physical dependency, it does not capture the full complexity of residents’ feeding needs, including cognitive and behavioral aspects. Additionally, multiple statistical tests were conducted without formal correction for multiple comparisons, which may increase the risk of type I error; however, most associations were significant at conservative levels (*p* ≤ 0.01).

The findings should be interpreted cautiously, and future studies should incorporate larger, multi-site samples, and more comprehensive measures of functional and feeding-related dependency, to strengthen external validity and allow for more robust statistical examinations.

## 6. Conclusions

This exploratory study contributes to a growing body of literature examining mealtime support from the perspective of DSPs in residential settings for individuals with IDD. While DSPs reported high levels of positive feelings regarding work efficacy, negative feelings such as stress and workload were significantly associated with contextual resident-related, institutional and environmental factors, and residents’ physical dependency. These findings suggest that mealtime experiences are shaped less by individual caregiver characteristics and more by relational and organizational conditions. Improving nutritional care in long-term IDD settings, therefore, requires not only attention to clinical feeding practices but also to the environment of care, which emerged as a key factor in shaping caregivers’ emotional experiences. Structural support, environmental adaptation, and communication-focused approaches may reduce DSPs strain and enhance sustainable, person-centered mealtime assistance. For example, simple environmental modifications such as appropriate lighting, a pleasant and inviting dining space, appealing food aromas, and a varied menu may enhance mealtime experiences. Although the study was conducted in a single residential facility, the multilevel determinants identified may be relevant to individuals with diverse disabilities and varying levels of dependency across different supported living arrangements. Future multi-site studies with larger samples are warranted to confirm and extend these findings.

## Figures and Tables

**Table 1:** Descriptive characteristics of attitude’s domains.

Factors	Mean (SD)	Range	α
Mealtime environment	3.24 (0.45)	2.50–4.25	0.63
Communication with residents	4.31 (0.62)	3.00–5.00	0.87
Accessibility and adaptability of the feeding environment	4.36 (0.48)	3.50–5.00	0.89
Institutional support	3.53 (0.94)	1.00–5.00	0.87
Negative feelings about job efficacy	3.91 (0.69)	2.00–5.00	0.73
positive feelings about job efficacy	4.64 (0.48)	3.67–5.00	0.74

## Data Availability

The data presented in this study are available upon request from the corresponding author due to privacy and confidentiality restrictions.

## Abbreviations

The following abbreviations are used in this manuscript:

ADL	Activity of Daily Living
IDD	Intellectual and Developmental Disabilities
DSP	Direct Support Professional
